# Upward Shifts in the Internal Representation of Frequency Can Persist Over a 3-Year Period for Cochlear Implant Patients Fit With a Relatively Short Electrode Array

**DOI:** 10.3389/fnhum.2022.863891

**Published:** 2022-03-25

**Authors:** Michael F. Dorman, Sarah C. Natale, Jack H. Noble, Daniel M. Zeitler

**Affiliations:** ^1^College of Health Solutions, Speech and Hearing Science, Arizona State University, Tempe, AZ, United States; ^2^Department of Electrical Engineering & Computer Science, Vanderbilt University, Nashville, TN, United States; ^3^Otolaryngology, Virginia Mason Medical Center, Seattle, WA, United States

**Keywords:** cochlear implant, single-sided deafness, sound quality, neural plasticity, neural prosthesis

## Abstract

Patients fit with cochlear implants (CIs) commonly indicate at the time of device fitting and for some time after, that the speech signal sounds abnormal. A high pitch or timbre is one component of the abnormal percept. In this project, our aim was to determine whether a number of years of CI use reduced perceived upshifts in frequency spectrum and/or voice fundamental frequency. The participants were five individuals who were deaf in one ear and who had normal hearing in the other ear. The deafened ears had been implanted with a 18.5 mm electrode array which resulted in signal input frequencies being directed to locations in the spiral ganglion (SG) that were between one and two octaves higher than the input frequencies. The patients judged the similarity of a clean signal (a male-voice sentence) presented to their implanted ear and candidate, implant-like, signals presented to their normal-hearing (NH) ear. Matches to implant sound quality were obtained, on average, at 8 months after device activation (see section “Time 1”) and at 35 months after activation (see section “Time 2”). At Time 1, the matches to CI sound quality were characterized, most generally, by upshifts in the frequency spectrum and in voice pitch. At Time 2, for four of the five patients, frequency spectrum values remained elevated. For all five patients F0 values remained elevated. Overall, the data offer little support for the proposition that, for patients fit with shorter electrode arrays, cortical plasticity nudges the cortical representation of the CI voice toward more normal, or less upshifted, frequency values between 8 and 35 months after device activation. Cortical plasticity may be limited when there are large differences between frequencies in the input signal and the locations in the SG stimulated by those frequencies.

## Introduction

The neural targets for electrical stimulation provided by a cochlear implant (CI) are cell bodies in the spiral ganglion (SG). The cell bodies are organized in tonotopic fashion (Stakhovskaya et al., 2007) with cell bodies that respond to high frequencies located near the place of electrode insertion and cell bodies responding to lower frequencies occurring further along the spiral toward the apex. Given this, it is reasonable to suppose that the sound quality of speech elicited by stimulation from a relatively long electrode array would differ from the sound quality elicited *via* a shorter array due to the lower frequencies stimulated by the longer array.

Two of the principal acoustic factors that determine a listener’s speech percept are the formant frequencies that reflect the resonant frequencies of a speaker’s vocal tract and the fundamental frequency of the voice or voice pitch (F0). A speaker’s formant frequencies are constrained by vocal tract geometry. A relatively short vocal tract will have higher formant frequencies than a longer vocal tract (Fant, 1970). Speech transmitted *via* a short electrode array and relatively high SG frequencies will sound more like speech produced *via* a short vocal tract than speech produced *via* a longer tract. Because vocal tract length is related to height (Fitch and Giedd, 1999), signals upshifted in frequency can sound as if they were produced by a relatively small person (Smith and Patterson, 2005).

The fundamental frequency of the voice (F0) can also influence the perception of a speaker’s characteristics. On average, men have lower F0 than women due to greater vocal cord mass. For a given vocal tract configuration, a high F0 will lead to the perception of a smaller person than a low F0 (Smith et al., 2005; Smith and Patterson, 2005).

When voiced speech is processed *via* CI hardware, F0 is the primary modulation in low frequencies. Eddington et al. (1978) reported that the pitch of a low-frequency sinusoid increased as that sinusoid was directed to electrodes toward the base, or high frequency portion, of the cochlea. Although subsequent research has documented several constraints on this outcome (e.g., Adel et al., 2019), it is likely that F0 will sound at least slightly higher *via* a shorter electrode array than a longer array. With a longer array, the rate/place mismatch can be minimized and a low-frequency rate can elicit an appropriate low-frequency pitch (e.g., Rader et al., 2016).

A speech signal with both an upshifted formant pattern and F0 may sound as if it were produced by one of the small-people actors, the Munchkins, in the 1930’s American film, *The Wizard of Oz*. Research conducted with single-sided deaf listeners fit with a cochlear implant (SSD-CI) indicates that this is often the case (Dorman et al., 2019a,b).

Dorman et al. (2019a) tested single-sided deaf (SSD) patients fit with either a 28 mm electrode array or a 18.5 mm array. The patients judged the similarity of a clean signal (a male-voice sentence) presented to their CI ear and candidate, CI-like signals presented to their normal-hearing (NH) ear. The signals to the NH ear were altered with the goal of creating a signal that matched the sound of the CI. The patients fit with the 28 mm electrode array were tested in a baseline (standard programming) condition and conditions in which the most apical, i.e., lowest frequency, electrode (E1) and both E1 and E2 were turned off and the input reallocated to the remaining more basal, or higher frequency, electrodes. In the E1-off and E1/E2-off conditions, matches to CI sound quality were characterized by an upshift of the frequency spectrum (formant frequencies) and/or increases in voice pitch (F0).

The two patients fit with 18.5 mm electrodes received an additional condition. In this condition, a current-steered virtual electrode (Wilson et al., 1994) whose pitch was lower than that of the most apical physical electrode, was added to the program. In the baseline condition, matches to CI sound quality were characterized by spectral upshifts for both patients. This is in contrast to the matches from the 28 mm electrode patients who did not need spectral upshifts to match CI sound quality in the baseline condition. Critically, when using the program which included the virtual, supra-apical electrode, the patients matched to signals with lower spectral frequency content than in the baseline condition. Together, the results suggest that CI sound quality is influenced by the lowest frequencies stimulated *via* an electrode array (see, also, Dorman et al., 2020).

The patients fit with 18.5 mm electrode arrays had 6 months or less experience with their CI. With more CI experience, it is possible that the perceptual upshifts in spectrum and/or F0 experienced by these patients would be reduced. One factor that could underlie this possibility is a reduction in electrode place-pitch over time (e.g., Reiss et al., 2007; McDermott et al., 2009). However, Tan et al. (2017) point out that not all studies have found such a shift with experience (see, also, Marozeau et al., 2020). Another factor that could underlie this possibility is a higher-level process, i.e., normalization–the essential property of the cortical, speech-processing neuro-architecture that allows signals with different acoustic signatures to be categorized as the same phonetic segment or as belonging to the same speaker (Johnson, 2005; von Kriegstein et al., 2010).

In this report, we tested five SSD-CI patients fit with 18.5 mm electrode arrays at two time points (Time 1 mean = 8.3 months vs. Time 2 mean = 34.8 months) following device activation. Our interest was to determine whether the perceived upshifts in frequency spectrum or F0 at Time 1 were reduced or eliminated at Time 2.

At the time of device activation, it was not practical to obtain sound quality matches using the procedure described above. Instead, patients were asked to provide, from memory, descriptions of sound quality. Patients were also asked to provide descriptions of sound quality just before the second sound-quality match (see section “Time 2”). At issue in this condition was whether the descriptions of sound quality would correspond to the matches to sound quality. For example, if a patient indicated that speech sounded “normal” at Time 2, then would the sound quality matches contain few, or no, altered signal components?

## Materials and Methods

### Participants

**FIGURE 1:**
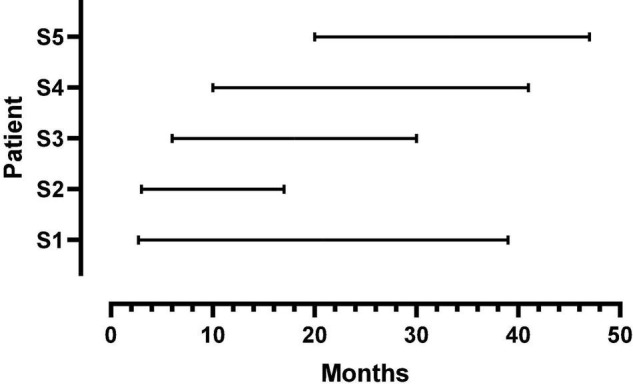
Duration of CI listening experience at first and second test points for each patient. The beginning and endpoint of each line indicates the first and second test times, respectively.

Five female listeners fit with Advanced Bionics Naida Q90 processors and 18.5 mm, mid-scalar, electrode arrays participated in this study. Biographical data are shown in Table 1. The mean pure tone average (0.5, 1.0, 2.0 kHz) in the not-implanted ear was less than 20 dB for each patient. The group mean age was 41 years with a range of 26–52 years at the time of the first test. The duration of deafness before implant ranged from 0.22 to 8.2 years. As shown in Figure 1, at Test 1 mean CI experience was 8.3 months with a range from 2.7 to 20 months. At Test 2, the mean CI experience was 34.8 months with a range of 17–47 months. Data logging indicated a mean duration of CI use per day of 11.5 h with a range of 8.8–13.8 h.

**TABLE 1:** Selected biographical data for patients.

Subject ID	Age at time of test 1 (years)	Duration deaf (years)	Insertion Depth (degrees)	SG frequency at most apical electrode (Hz)	AzBio quiet (percent correct) time 1	AzBio quiet (percent correct) time 2
1	38	1.7	409	650	76	62
2	26	2.7	387	750	65	72
3	49	3.5	419	620	77	58
4	40	0.22	395	680	76	63
5	52	8.2	340	890	71	62

The mean AzBio in quiet score at Time 1 was 73 percent correct with a range of 65–77 percent correct. The mean score at Time 2 was 63 percent correct with a range of 62–72 percent correct. Gifford et al. (2018) reported, for a large sample of CI patients, a mean score of 63 percent correct on this test.

**FIGURE 2:**
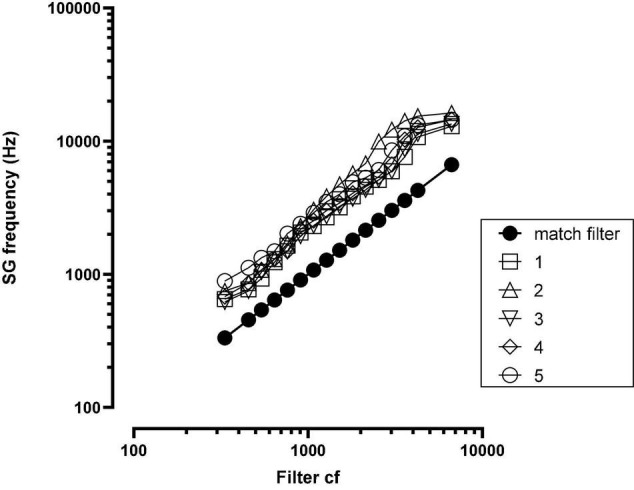
Spiral ganglion (SG) frequency at each electrode for each patient. The solid black function (match filter) indicates the SG frequency matched to the filter cf. SG = spiral ganglion; cf = center frequency; numbers in legend = patient number.

Post-implant, CT imaging of the cochlea indicated a mean insertion angle of 390 degrees with a range of 340–419 degrees. The SG frequency at the most apical electrode, calculated by the method described in Noble et al. (2013), was 718 Hz with a range of 620–890 Hz. Figure 2 shows, for each patient, the SG frequency near each electrode.

In the following sections, we describe our test signals and test methods. Rather than refer to previous papers, we “recycle” (Science 2 July 2021, 8–9) our previous descriptions with changes necessitated by upgrades to signal processing algorithms.

### Test Signals

Two, male-voice sentences from the CUNY sentence corpus were used for testing: “Do you like camping?” and “The sun is finally shining.” The sentences were first synthesized using the STRAIGHT (Kawahara et al., 2001) algorithm so that other manipulations could be implemented efficiently. These sentences were chosen because the synthesized version and the natural version were nearly indistinguishable. One sentence was used for each subject’s test session. The mean F0 for the sentence, “Do you like camping?” was 139 Hz with a range of 114–188 Hz. The mean F0 for the sentence, “The sun is finally shining,” was 129 Hz with a range of 86–200 Hz.

Custom-built software produced changes in the acoustic characteristics of each sentence in order to create candidate sentences for the NH ear. Sound changing operations could be implemented, *via* an on/off toggle, singly or in any combination. At output, signal modifications were implemented in the order described below.

The mean fundamental frequency of the voice (F0) could be increased or decreased and the F0 contour (i.e., the intonation contour) could be flattened in steps from 100 to 0% of the normal extent. The flattening algorithm kept the mean F0 the same as in the original file but altered the end points. A completely flattened F0 contour, i.e., a monotone, commonly elicits the percept of a robotic talker. A large increase in F0 *per se* elicits a Mickey Mouse™ percept.

Formant frequencies could be shifted over the range −50z to + 300 Hz. In our implementation, the difference in frequency between formants was maintained and the whole spectrum was shifted up or down in frequency linearly. Unpublished research from our laboratory using normal hearing listeners indicates that a 125 Hz upshift in spectrum, without a change in F0, produces a voice quality similar to that produced by the Munchkin characters in *The Wizard of Oz* (Dorman et al., 2019b).

Spectral peaks could be broadened and spectral peak-to-valley differences reduced in a simulation of the effects of poor frequency selectivity (algorithm modeled after Baer and Moore, 1993). For a synthetic-vowel test signal, with smear = 0, the F1 spectral peak-to-valley amplitude difference was 23.9 dB; with smear = 5 (moderate smear), the difference was 17.1 dB and with smear = 10 (maximum smear), the difference was 11.8 dB. Because the amplitude of spectral peaks for voiced sounds falls with frequency, at high degrees of broadening signals have a low-pass characteristic and sound muffled. At high levels of broadening, a low level of a static-like sound was introduced. This sound quality proved useful when patients asked for a “buzzy” signal.

Noise and sine vocoders could be implemented with 4–12 channels (see Dorman et al., 2017). A noise vocoder has a hissy quality and a sine vocoder has an electronic “whine.” Both types of vocoder outputs could be combined with a non-vocoded signal.

A slight frequency and amplitude shift over time was implemented by creating a signal whose sample rate was 0.1% lower than that of original signal and then combining the two signals. Perceptually the combined signal sounded slightly comb filtered. Our implementation was only one of many possible implementations of a “flange” operation. This operation was suggested to us by a patient who played guitar and who said that his implant sounded “flanged.”

Speech signals could be given a metallic sound quality by altering resonances and ring times. Because struck metal objects often have inharmonically related resonant frequencies and long ring times, a filter was constructed using a bank of sharp, inharmonically related resonances in combination with a bandpass filter. The resonant frequencies were *f* = (442, 578, 646, 782, 918, 1054, 1257, 1529, 1801, 2141, 2549, 3025, 3568, 4248 Hz).

Signals could be low-, high- and band-pass filtered using 6th order Butterworth filters with variable corner frequencies. Low-pass and band-pass filtering produce a muffled sound quality. Band-passed signals commonly sound as if they are farther away than wide-band signals. Filtering can also create a “tinny” sound quality and the sound quality of a “transistor AM radio.” Both are common descriptions of CI sound quality.

### Procedure

The procedure used for patient testing is shown in videos in Dorman Dorman et al. (2019a,b) and Dorman et al. (2020) and is described below. For all conditions, signals were delivered to the CI *via* a direct connect cable and signals were delivered to the NH ear *via* an insert receiver (ER3-A). The patient and the experimenter sat at a table facing each other. The second author operated the sound mixing console. A clean signal was delivered to the CI first and then to the NH ear. The experimenter asked the patient how the signal to the NH ear should be changed to match the sound in the CI ear. A list of 64 audio terms was given to patients to help with descriptions of sound quality (Dorman et al., 2020).

For example, if the patient said that the signal to the NH ear should be “higher” then the pitch shift or formant shift operations, or both, were implemented. The experimenter continued to manipulate these dimensions until the patient said that the pitch was very close to that of the CI. We note that patients commonly describe both an increase in pitch and in formant frequencies as an increase in “pitch.” Operationally and perceptually the effects of the manipulations are quite different.

Once the patient was satisfied that the pitch was near that of the CI, then the experimenter asked if the pitch contour, or intonation, was correct. If the answer was “no,” the experimenter would begin to flatten the contour. This continued until the patient said that the contour was “very close” to that of the contour produced by the CI.

This process, asking the patient what needed to be changed and then altering the signal, continued until the patient said that the match was “very close” and/or the parameter set had been exhausted. At this point, the patient was asked to rate the similarity of the signal presented to the NH ear relative to that of the CI on a 10 point scale with 10 being a complete match.

### Questionnaire

Within a month before the second test, patients were sent *via* email a form on which they were asked to describe the sound quality of their implant (i) just after fitting and (ii) “now.” A list of sound quality attributes was provided for reference (Dorman et al., 2020). The patients were also asked: Did you have to relearn familiar voices or did you adapt quickly? The answer to this question bears on the issue of the amount of distortion present in the signal shortly after device activation.

All procedures were approved by the Arizona State University Review Board for the Protection of Human Subjects (IRB).

## Results

**FIGURE 3:**
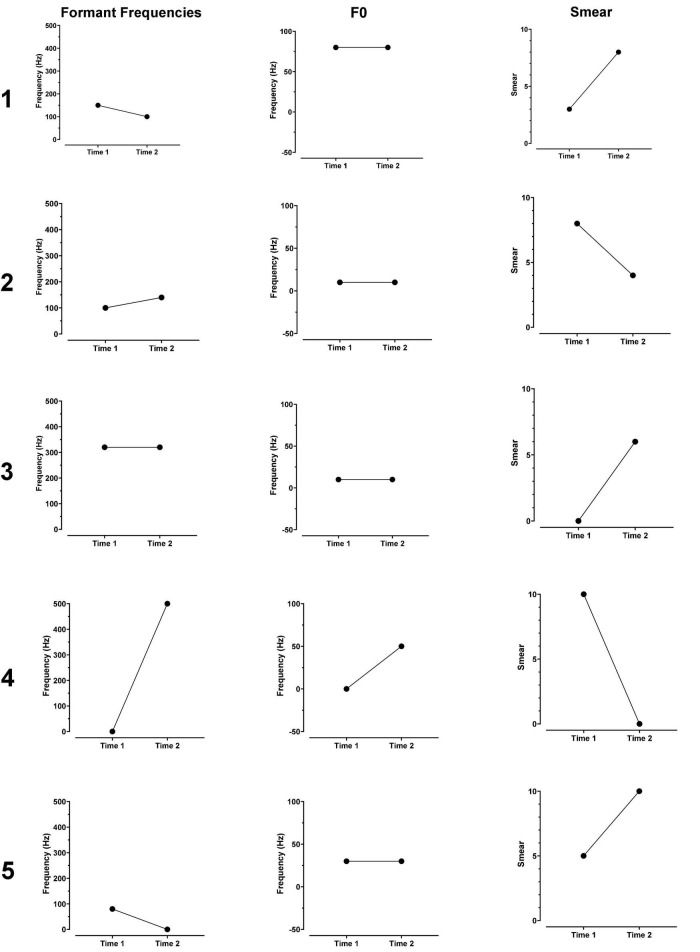
Changes in formant frequencies, F0 and smear, relative to a clean signal, at Time 1 and Time 2. Numbers at the far left of each figure indicate patient number.

The parameter values used to create an approximation to CI sound quality at Time 1 and Time 2 are listed in Table 2 for each patient. Figure 3 shows the changes in formant frequencies, F0 and smear, relative to a clean signal, at Time 1 and Time 2.

**TABLE 2:** Stimulus alterations needed to match CI sound quality at Time 1 and Time 2.

	First visit	Second visit	Insertion angle and SG frequency	Match rating
Patient 1	***@ 2.7 m*** Formant: + 150 Hz; F0: + 80 Hz; F0 contour: 100%; Smear: Low; BP 400–4000 Hz; Flanger on; Metallic.	***@ 39 m*** Formant: + 100 Hz; F0: + 80 Hz; F0 contour: 25%; Smear: High; HP 400 Hz; Flanger off; Metallic.	409° 650 Hz	1st = 9.2 2nd = 9.8
Patient 2	***@ 3 m*** Formant: + 100 Hz; F0: + 10 Hz; F0 Contour: 90%; Smear: High; Flanger on.	***@ 17 m*** Formant: + 140 Hz; F0: + 10 Hz; F0 contour: 70%; Smear: Low; Flanger off.	387° 750 Hz	1st = 9.8 2nd = 9.9
Patient 3	***@ 6 m*** Formant: + 320 Hz; F0: + 10 Hz; F0 contour: 100%; Smear: None.	***@ 30 m*** Formant: + 320 Hz; F0: + 10 Hz; F0 contour: 100%; Smear: Medium.	419° 620 Hz	1st = 9.7 2nd = 9.0
Patient 4	***@ 10 m*** Formant: no change; F0: no change; F0 contour: 25% Smear: High; BP 400–2000 Hz; Noise VC: 10 channel; Flanger: on.	***@ 41 m*** Formant: + 500 Hz; F0: + 50 Hz; F0 contour: 0%; Smear: None; BP 400–5000 Hz; Noise VC:12 channel; Flanger: off.	395° 680 Hz	1st = 7 2nd = 8
Patient 5	***@ 20 m*** Formant: + 80 Hz; F0: + 30 Hz, F0 contour: 100%; Smear: Medium; BP 400–1500 Hz; Flanger: on	***@ 47 m*** Formant: no change; F0: + 30; F0 contour:100%; Smear: Max; High pass 400 Hz; Flanger: off	340° 890 Hz	1st = 8 2nd = 9

### Time 1

The data reported below are relevant to the question of whether formant frequencies or F0 are upshifted after 2.7–20 months experience with a CI.

Ratings of the similarity of the modified clean signals to the CI signals ranged from 7 to 9.8 with a mean of 8.7. Patients 1, 2, and 3 produced ratings of 9.2, 9.8, and 9.7, respectively. These ratings indicate very close matches the sound of the patients’ CIs. Additionally, these three patients had the least experience with their CIs, i.e., 2.7, 3, and 6 months. For these reasons, the matches produced by Patients 1, 2, and 3 are described first.

To approximate the sound of their CI, all three patients needed an increase in formant frequencies. Values ranged from 100 to 320 Hz. All three patients asked for an increase in F0. Two wanted a small increase of 10 Hz while the third wanted a large increase of 80 Hz. Two of the three patients indicated better matches with smearing or with a flanged signal.

For the remaining two patients, matches were less close to the sound of their CI (match scores of 7 and 8) than for the patients described above. One patient required no change in formant frequencies or F0 for a match while the other required an 80 Hz shift in formant frequencies and a 30 Hz shift in F0. Both patients need smearing and flanging to approximate the sound of their CI.

Overall, four of the five listeners needed an increase in formant frequencies and an increase in F0 to match the sound of their CI. In addition, four of the five listeners needed both the flanging operation and the smearing operation. Three of the five need a reduction of signal bandwidth.

### Time 2

At issue in this data set is whether the approximations show fewer, or smaller magnitude, deviations from a clear signal than at Time 1. Data from Patients 1–3, with match ratings of 9.8, 9.9, and 9.0, are described first. CI experience, at Time 2, ranged from 17 to 39 months in contrast to 2.7–6 months at Time 1.

At Time 2, formant values remained elevated for the three patients–increasing slightly for one patient, remaining the same for one patient and decreasing slightly for one patient. Values of F0 did not change and remained elevated. Smear increased for two of the three and decreased for one. A metallic sound quality at Time 1 for Patient 1 remained at Time 2.

Results were mixed for Patients 4 and 5. For Patient 4, increases in both formant frequencies (500 Hz) and in F0 (50 Hz) were needed to match CI sound quality. For Patient 5 a decrease in formant frequencies was needed. However, an elevated F0 (30 Hz–the same as at Time 1) remained.

### Questionnaire Data

The descriptions of sound quality near the time of device activation and near the time of the second sound quality match are shown in Table 3.

**TABLE 3:** Descriptions of sound quality near time of activation and at Time 2.

Subject ID	Qualities near activation	Experience months (“now”)	Qualities now	Did you have to relearn familiar voices or did you adapt quickly?
1	Chipmunk-like, congested, computer-like, distorted, dull, far-away, grainy, high-pitched, mickey mouse-like, muffled, nasal, treble-y	39	Clear, crisp, detailed, dynamic, full, lush, normal, rich	Recognizable
2	Bass-y, computer-like, edgy, grungy, metallic	17	Clear, normal, rich, tinny	Relearn
3	Aggressive, chipmunk-like, crisp, distorted, metallic, mickey mouse-like, tinny	30	Aggressive, boom-y, normal, spacious	Recognizable
4	Aggressive, blurred, chipmunk-like, congested, computer-like, Darth Vader-like, distorted, grainy, grungy, nasal, steely	41	Blurred, congested, computer-like, Mickey-mouse like	Relearn
5	Blanketed, chipmunk-like, computer-like, distorted, grainy, metallic, mickey mouse-like, muffled, Munchikin-like, steely, tinny	47	Blanketed, computer-like, dynamic, normal, smooth, warm	Relearn

Following device activation, common descriptors of sound quality included chipmunk-like, Mickey Mouse-like, tinny, metallic and high pitched. Computer-like and distorted were also common. Three of the five patients indicated that they had to relearn the sound of different voices.

At Time 2 (following 17–47 months of CI experience), the frequency of use of all of the terms listed above was reduced. Descriptors such as clear and normal appeared for patients 1, 2, 3, and 5. For Patient 4, the descriptors were similar near the time of activation and after experience.

## Discussion

In the current study, our principal interest was to examine, for patients fit with a relatively short electrode array, whether perceived upshifts in frequency spectrum and/or F0 at approximately 8 months following device activation changed over time toward a smaller upshift.

### Difference Between Spiral Ganglion Frequencies and Filter Frequencies

As shown in Figure 1, there were large offsets between the filter frequencies in the speech processor and SG place frequencies. Energy extracted from the speech signal was delivered to SG place frequencies that were between one and two octaves higher than the frequencies in the signal. If there were no factors to fully offset these upshifts, then patients should have created matches to CI sound quality using upshifted frequency components.

### Similarity of Approximations to Cochlear Implant Sound Quality

In order to evaluate the hypothesis of a change in sound quality over time, approximations to CI sound quality need to be very similar to CI sound quality. Patients 1, 2, and 3, on average, gave scores of 9.6 on the scale of 1–10 to the approximations at both Test 1 and Test 2. Because of this, it is reasonable to focus initially on the data from these three patients. To provide an aural example of the sound of these patients’ CIs, audio files from Patient 1 (Time 2, match score = 9.8), Patient 2 (Time 2, match score = 9.9) and Patient 3 (Time 1, match score = 9.7) are included in Supplementary Digital Material.

### Changes in Sound Quality Matches Over Time

At Test 1, both formant frequencies and F0 were upshifted. The shifts in formant frequencies were 150, 100, and 320 Hz for patients 1, 2, and 3, respectively. The shifts in F0 were 80, 10, and 10 Hz, respectively. At Test 2, more than 2 years later, formant frequencies and F0 frequency remained upshifted.

Patient 5 produced a match score of 9 at Test 2 and was the only patient whose approximations contained upshifted formants at Test 1 but not at Test 2. However, a 30 Hz increase in F0, found at Test 1, remained at Test 2 indicating the continuation of an upshifted percept. In contrast, Patient 4 needed no upshift in formant frequencies or F0 at Test 1 but required very large increases to both at Time 2.

Overall, the sound quality matches provide very little support for the hypothesis that cortical plasticity alters the representation of the CI voice toward less upshifted frequency values over the time intervals examined in this study. This outcome is consistent with a conclusion of Tan et al. (2017), i.e., that cortical plasticity, in terms of an apical shift in electrode place pitch, “is not always sufficient to overcome large frequency-position mismatches.”

### Changes in Sound Quality Descriptions Over Time

As noted in the introduction, it was not practical to obtain sound quality matches near the time of device activation. For that reason, we asked the patients to describe from memory the sound qualities they experienced at that time. As shown in Table 3, a common descriptor was “distorted.” Descriptors invoking the sensation of a high-pitched signal were common. These included chipmunk-like, high-pitched, Mickey Mouse-like, treble-y and metallic. Computer-like was also common. Although these descriptions of sound quality could have been influenced by recall bias (Bradburn et al., 1987), the descriptions are consistent with clinicians’ reports of patient descriptions of sound quality obtained near the time of device activation.

Three of the five patients indicated that they had to relearn the sound quality of familiar voices. This is consistent with an initial, abnormal representation of voice. It is of interest that the two patients who said that familiar voices were recognizable early after activation (Patients 1 and 3) were the two patients with the least upshifted SG values relative to filter frequencies.

As expected, at the time of the second test the descriptions of sound quality changed for most of the patients. For four of the five, the descriptors suggested a better sound quality including “normal.” Other descriptors that suggested better sound quality included clear, warm, rich, spacious, detailed, dynamic. For Patient 4 the descriptors at Time 2 suggested less change from the time of device activation. These descriptors included: blurred, congested, computer-like and Mickey Mouse-like.

Curiously, the choice of the descriptor “normal” could be paired with descriptors suggesting that the percept was not normal. These descriptors included tinny, boom-y, blanketed and computer-like. Only Patient 1 chose descriptors that were completely consistent with normal sound quality. That said, we turn now to a comparison of sound quality descriptions and sound quality matches at Test 2 and begin with Patient 1.

### Feel vs. Real

As shown in Table 4, there could be large differences between sound quality descriptions and sound quality matches at Test 2. For Patient 1, the descriptions of sound quality included the terms clear, crisp, detailed, dynamic, full, lush, normal and rich. Consider now the sound quality match (a rating of 9.8 out of 10): F0 = + 80 Hz; F0 contour = 25% of normal; Formant = + 100 Hz; Smear = high; Bandwidth = HP 400 Hz; Metallic. This signal is far from normal in multiple dimensions. Patients 2, 3, and 5 also used the descriptor “normal” and, like Patient 1, matched to signals with upshifted F0 and/or formant frequencies and high degrees of smear.

**TABLE 4:** Sound quality descriptions and sound quality matches at Time 2.

Subject #	Experience months	Description	Match
1	39	Clear, crisp, detailed, dynamic, full, lush, normal, rich	Formant + 100 Hz; F0 + 80 Hz; F0 contour 25%; Smear High; HP 400 Hz; Metallic
2	17	Clear, normal, rich, tinny	Formant + 140 Hz; F0 + 10 Hz; Pitch contour 70%; Smear Medium
3	30	Aggressive, boom-y, normal, spacious	Formant + 320 Hz; F0 + 10 Hz; Smear Medium
4	41	Blurred, congested, computer-like, Mickey-mouse like	Formant + 500 Hz; Pitch + 50 Hz; Pitch contour 0%; Smear None; BP 400–5000 Hz; Noise VC 12 channel
5	47	Blanketed, computer-like, dynamic, normal, smooth, warm	Formant no shift; Pitch + 30; Pitch contour 100% Smear High; High pass 400 Hz

### Does Normal=Familiar?

What might account for use of the descriptor “normal” when the sound quality matches were far from normal? At device activation and for some time after, perhaps long after, the voice of a CI is not the voice that was previously associated with a familiar speaker. In this sense, the CI voice is unfamiliar, i.e., it is not matched to the internal representation of the speaker’s voice. With CI experience, including pairing CI sound with a speaker’s face, the voice will become familiar – even though it is altered. Familiar and unfamiliar voices elicit different patterns of connectivity between voice recognition regions in the anterior and posterior right, superior temporal sulcus (STS) (e.g., von Kreigstein and Giraud, 2004), i.e., they are processed differently. It may be that the experience of “normal” for some patients is a byproduct of the change from processing a speech signal as unfamiliar to processing that signal as familiar. Researchers should be wary of patient reports of normal sound quality. For reviews of the neural substrates of, and a conceptual model for, voice processing see von Kriegstein et al. (2006, 2010).

### Speech Understanding

At the time of the first test, all of the patients had speech-understanding scores in quiet that were at or above the mean score of 63 percent correct reported by Gifford et al. (2018) for a very large sample. Thus, the frequency upshifts and other distortions in the representation of the signal found for these patients when matching voice quality did not have a large negative impact on speech understanding.

At the time of the second test, speech understanding did not increase as would be expected if the cortex responding to the NH ear tried to bootstrap, i.e., tried to improve, the signal from the CI ear. It appears that, over the time interval studied here, the cortex responding to the signal from the NH ear treats the CI signal with benign neglect.

### Limitations

The sample size was very small and that brings into consideration the many, and often discussed, problems associated with small samples (e.g., Button et al., 2013).

The range of electrode insertion angles for our listeners was from 340 to 419° with a median angle of 395°. Landsberger et al. (2015), for a sample of 30 patients, reported a range between 257 and 584° and a median angle of 391°. Thus, our sample, while small, was not unrepresentative.

We did not assess cognitive function in our patients. Both bottom-up and top-down processes contribute to speech understanding in CI patients (e.g., Zhan et al., 2020; Moberly et al., 2021). The contribution of top-down factors to the recognition of voice or CI sound quality is unexplored.

## Conclusion

For SSD-CI patients with electrode-array insertion angles between 340 and 419 degrees, the internal frequency representation of the speech signal can remain upshifted several years after device activation. This indicates that cortical plasticity can be limited when there are large differences between the frequencies in the input signal and the place of electrical stimulation in the SG.

## Data Availability Statement

The original contributions presented in the study are included in the article/Supplementary Material, further inquiries can be directed to the corresponding author.

## Ethics Statement

The studies involving human participants were reviewed and approved by the Institutional Review Board, Arizona State University. The patients/participants provided their written informed consent to participate in this study.

## Author Contributions

MD was responsible for the initial draft and final editing of the manuscript. SN was responsible for patient testing and data collection. JN was responsible for the analysis of the high-resolution and computerized tomography (CT) scans that determined electrode location in the cochlea. DZ was the CI surgeon. All authors contributed to the manuscript and all approved the submitted version.

## Conflict of Interest

MD was a consultant for MED-EL and Advanced Bionics. DZ was a consultant for Advanced Bionics. A portion of SN’s salary was supported by MED-EL. The remaining author declares that the research was conducted in the absence of any commercial or financial relationships that could be construed as a potential conflict of interest.

## Publisher’s Note

All claims expressed in this article are solely those of the authors and do not necessarily represent those of their affiliated organizations, or those of the publisher, the editors and the reviewers. Any product that may be evaluated in this article, or claim that may be made by its manufacturer, is not guaranteed or endorsed by the publisher.
